# Farmers’ Rights and Digital Sequence Information: Crisis or Opportunity to Reclaim Stewardship Over Agrobiodiversity?

**DOI:** 10.3389/fpls.2021.686728

**Published:** 2021-08-13

**Authors:** Rachel Wynberg, Regine Andersen, Sarah Laird, Kudzai Kusena, Christian Prip, Ola Tveitereid Westengen

**Affiliations:** ^1^Department of Environmental and Geographical Science, University of Cape Town, Cape Town, South Africa; ^2^Fridtjof Nansen Institute, Lysaker, Norway; ^3^People and Plants International, Bristol, VT, United States; ^4^Department of Research and Specialist Services, National Genebank of Zimbabwe, Harare, Zimbabwe; ^5^Department of International Environment and Development Studies (Noragric), Norwegian University of Life Sciences, Aas, Norway

**Keywords:** digital sequence information (DSI), access and benefit sharing (ABS), stewardship, ownership, farmers’ rights

## Abstract

Contestations about the way in which digital sequence information is used and regulated have created stumbling blocks across multiple international policy processes. Such schisms have profound implications for the way in which we manage and conceptualize agrobiodiversity and its benefits. This paper explores the relationship between farmers’ rights, as recognized in the International Treaty on Plant Genetic Resources for Food and Agriculture, and the dematerialization of genetic resources. Using concepts of “stewardship” and “ownership” we emphasize the need to move away from viewing agrobiodiversity as a commodity that can be owned, toward a strengthened, proactive and expansive stewardship approach that recognizes plant genetic resources for food and agriculture as a public good which should be governed as such. Through this lens we analyze the relationship between digital sequence information and different elements of farmers’ rights to compare and contrast implications for the governance of digital sequence information. Two possible parallel pathways are presented, the first envisaging an enhanced multilateral system that includes digital sequence information and which promotes and enhances the realization of farmers’ rights; and the second a more radical approach that folds together concepts of stewardship, farmers’ rights, and open source science. Farmers’ rights, we suggest, may well be the linchpin for finding fair and equitable solutions for digital sequence information beyond the bilateral and transactional approach that has come to characterize access and benefit sharing under the Convention on Biological Diversity. Existing policy uncertainties could be seized as an unexpected but serendipitous opportunity to chart an alternative and visionary pathway for the rights of farmers and other custodians of plant genetic resources.

## Introduction

For the first time in its history, a Governing Body session of the International Treaty on Plant Genetic Resources for Food and Agriculture (ITPGRFA), meeting in Rome in November 2019, was finalized without a closing session. After several years of tough negotiations (e.g., Food and Agriculture Organization (FAO), 2019a), delegates had arrived with the hope that an enhanced multilateral system (MLS) for access and benefit sharing for plant genetic resources for food and agriculture (PGRFA) could be finalized and adopted. However, in an unprecedented outcome, negotiations collapsed and there was no plan for a further formal process to conclude these discussions.

Among the central questions stalling negotiations was whether “digital sequence information” (DSI) – meaning genetic or nucleotide sequence data^1^, in this context originating from PGRFA, should be included in the enhanced MLS. Vast amounts of DSI data are stored today in open access or open source databases and are used extensively by the scientific community for both basic and applied research, including the breeding and development of new plant varieties, as well as other biotechnology applications and products (Laird and Wynberg, 2018; AHTEG, 2020). Despite wide agreement that these databases are vital for biodiversity management and food security research, countries of the global South, led by the Africa Group, insisted that “failure to include DSI in the multilateral system would stall the deal as genetic material includes genetic information and sequencing, and Africa cannot agree to a system that will be unfit for purpose in the near future” (African Union, 2019). This rationale was based on the need for the compromise package to take scientific and technological advancements into consideration, and to ensure fair and equitable benefit sharing. Aligning with this position, the International Planning Committee for Food Sovereignty (IPC), a global platform described as representing more than 6,000 organizations and 300 million small-scale food producers and rural workers’ organizations and social movements, remarked that “DSI constitutes a socio-political issue, which if not dealt with now in its entirety, will jeopardize Farmers’ Rights to save, use, exchange and sell their seeds” (Muzurakis, 2019^2^).

These positions were not supported by many countries from the global North, linked largely to a concern that the inclusion of DSI in the MLS would restrict access to genetic sequence databases, impede scientific understanding and technological innovation, and curtail benefits arising from their use^3^. The ensuing deadlock at the beginning of the Governing Body meeting led to the suspension of plenary negotiations on the MLS, with the establishment of a small group that ultimately failed to reach consensus. A similar impasse over DSI has played out in several other policy processes under the auspices of the United Nations (UN), including the Convention on Biological Diversity (CBD) and its Nagoya Protocol on Access to Genetic Resources and the Fair and Equitable Sharing of Benefits Arising from their Utilization, the Pandemic Influenza Preparedness Framework of the World Health Organization, and the UN Convention on the Law of the Sea (Laird et al., 2020).

Schisms over DSI have profound implications for the way in which we both manage and conceptualize agrobiodiversity and its benefits. This paper aims to open questions about the relationship between farmers’ rights, as recognized in the ITPGRFA, and the dematerialization of genetic resources, given that crop genetic resources comprise both physical components, whereby plants contain functional units of heredity, and informational components, whereby the molecular basis for traits can be identified and sequenced (Halewood, 2013). In doing so, we draw on the analytical framework developed by Andersen (2006, 2016a, 2017) that uses the concepts of “stewardship” and “ownership” to elucidate different approaches and rifts related to agrobiodiversity management between proponents of farmers’ rights. The stewardship approach describes the idea that agrobiodiversity belongs to the common heritage of humankind and that it should be shared for the common good as part of the public domain. It was the dominant rationale throughout the history of agriculture until the advent of intellectual property rights (IPRs) and the subjecting of genetic resources to national sovereignty. The ownership approach evolved in the second half of the twentieth century, alongside the commercial use of genetic resources and advances in biotechnology, including IPRs to protect and promote inventions. Liberal policy formulation for agricultural development and a shift from publicly to privately funded research supported the parallel increase in privatizing genetic resources (Kloppenburg, 1988, 2004; Buhler et al., 2002; Brush, 2007; Andersen, 2008; Wynberg et al., 2018).

The history and rationales underlying the stewardship and ownership approaches to the governance of agrobiodiversity offer important lessons and insights for the development of policies to address DSI. In this article we argue that, given concerns raised under the ownership approach, the stewardship approach provides a more promising basis for addressing the equity issues associated with DSI under the ITPGRFA.

On this basis, we also explore whether the stewardship approach to farmers’ rights can open up new ways to understand and govern DSI, thereby centering and recognizing farmers as stewards and innovators of agrobiodiversity, equitably rewarding them for this contribution, securing their rights to participate in decision-making, and safeguarding any rights that farmers have to save, use, exchange and sell farm-saved seed, as set out in Article 9 of the ITPGRFA. In doing so, we examine the contradictions of a benefit-sharing system that is inextricably tied to the profits generated from seed sales, related inputs and associated IPRs, and which thus explicitly supports the enclosure of the commons and the commodification of genetic resources (van Dooren, 2008; West, 2012; Kloppenburg, 2014). Farmers’ rights, we suggest, may well be the linchpin for finding fair and equitable solutions for DSI beyond the bilateral and transactional approach that has come to characterize access and benefit sharing (ABS) under the CBD. As commentators suggest, this ABS approach has introduced concepts of “property, exclusivity and exclusion” to traditional agricultural communities, working to “erode the spirit and nature of Farmers’ Rights as a whole” (Brush, 2007; West, 2012). We also argue that the open access or open source nature of the 1.5 billion genetic sequences now included in the global dataset of the International Nucleotide Sequence Data Collaboration (INSDC)^4^ and other data repositories (Scholz et al., 2020) is antithetical to the bilateral models of ABS that have unfolded under the CBD over the past 30 years, but may well be aligned with the multilateral approach embraced by the ITPGRFA. Such approaches, we suggest, may offer potential solutions to “reconstitute the commons” (Kloppenburg, 2014) although their use requires careful attention to ensure alignment with, and enable protection of, the customary norms and practices of farmers and those conserving agrobiodiversity.

We begin the paper by describing how the management of plant genetic resources has transformed from a “stewardship” approach based on common heritage principles, to one that has become characterized by private ownership and the concentration of capital. We chart the history of the CBD and its Nagoya Protocol, situate these agreements in their political, economic and environmental contexts, and describe the contrasting multilateral approach adopted by the ITPGRFA. Locating the emergence of DSI within this milieu, we describe its intersection with farmers’ knowledge and the growing importance of genomic-based research. We then provide an analysis of the relationship between DSI and different elements of farmers’ rights, using the stewardship/ownership lens to compare and contrast implications for DSI governance. The paper concludes by setting out a number of options for finding fair and equitable solutions for DSI through a stewardship approach to farmers’ rights.

## From Stewardship to Ownership

### Historical Perspectives on Agrobiodiversity Governance in International Agreements

Throughout the history of agriculture, plant genetic resources have been managed based on “common heritage” principles, belonging to the public domain and not owned or otherwise monopolized by a single group or interest (Brush, 2007). This “stewardship” approach (Andersen, 2006, 2016a,b) has enabled farmers to continue as stewards and innovators of agrobiodiversity. Indeed, as Brush (2007, 1500) remarks, “common heritage is logical within farming communities where land and other natural resources are communally owned, seed is exchanged or shared, invention is collective, provenance is ambiguous, and natural and artificial selection are intertwined.”

The past eighty to ninety years, however, have witnessed a dramatic shift in the ways in which agrobiodiversity is both used and owned, with the stewardship approach for managing the use of and access to crop diversity coming under increasing, erosive pressure. As Kloppenburg (2004) notes, agricultural plant sciences have over time become increasingly subordinate to capital, shaping both the character of research and its products. Farmers have progressively been separated from the means of agricultural production such as seed, while the expansion of agribusiness and the global imposition of IPRs has led to a concentration in the ownership of land, seed and, now, genetic sequences (Kloppenburg, 2004, 2014; Desmarais, 2007; Clapp, 2018). Advances in science and technology have accelerated these transformations, enabling the emergence of a lucrative biotechnology industry, supported by a permissive IPR regime which, through the Trade Related Aspects of Intellectual Property Rights Agreement (TRIPs) of the World Trade Organization (WTO)^5^, adopted in 1994, has dramatically expanded the rights of companies to claim ownership over biodiversity-related innovations (Dutfield, 2000; Mytelka, 2000; Borowiak, 2004; Andersen, 2008).

As WTO members, most countries are now obliged to accommodate the TRIPs requirement for either patent protection or an effective *sui generis* (of its own kind) IPR system for plant varieties, which has been mostly Plant Variety Protection (PVP) under the International Union for the Protection of New Varieties of Plants (UPOV) convention.^6^ The UPOV convention has gradually strengthened the protection of plant breeders’ rights while, at the same time, other forms of IPRs on plant varieties and traits, such as patents and contract law, have become increasingly influential in agriculture and food production (Andersen, 2008; Haugen, 2015; Bjørnstad and Westengen, 2019).

An intensifying trend has been the ongoing consolidation of the seed, agrichemical and plant biotechnology industries, leading to the formation of ‘life science giants’ (Howard, 2016; Bonny, 2017). Over the past three decades, a series of mergers and acquisitions has created the “Big Six” – Monsanto, Bayer, Dupont, Syngenta, Dow, and BASF – all active in crop protection chemicals and, with the exception of BASF, also with strong positions in seed and new genetic technologies. A recent merger wave has reduced the number of major firms to just four (Bayer-Monsanto, DowDuPont/Corteva, ChemChina-Syngenta, BASF) that control the US$52 billion^7^ seed market (Clapp, 2018; Organisation for Economic Co-operation and Development [OECD], 2018). While precise valuations are difficult to calculate, the top four to five companies are estimated to control between 54 and 60% of global commercial seed sales (Maisashvili et al., 2016; Bonny, 2017; IPES-Food, 2017). This has resulted in the concentration of resources, plant breeding and seed supply in a limited number of hands and places, alongside growing fears of increased farmer and food dependency on a few big companies. Agreements such as UPOV and TRIPS have been integral to supporting these patterns of accumulation and privatization (van Dooren, 2008).

Such trends, combined with asymmetries in global patterns of seed commerce and exchange between lower income countries of the global South and higher-income, more industrialized nations of the global North, have been central to the “seed wars,” which characterized the long and arduous negotiations in the UN to develop global governance mechanisms for PGRFA (Mooney, 2011). An early milestone was reached when the International Undertaking on Plant Genetic Resources for Food and Agriculture was adopted in 1983, based on the principle that PGRFA were “the common heritage of mankind,” although this was later made conditional on the “sovereignty of the states over their plant genetic resources” (FAO resolution 3/91).

The shift away from a “common heritage” approach to genetic resources was further cemented with the 1992 adoption of the CBD, which affirmed national sovereignty over genetic resources, and linked the objectives of biodiversity conservation, sustainable use, and fair and equitable benefit sharing. Using their leverage as the main repositories of biodiversity, countries of the global South argued that in order to allow companies to access their biodiversity and associated traditional knowledge, the technologically rich industrialized countries should transfer technology and share benefits from biodiversity commercialization (Sanchez and Juma, 1994; Macilwain, 1998). This was a response in particular to the ongoing negotiations under the Uruguay Round of the General Agreement on Tariffs and Trade (GATT) which ultimately led to the adoption of the WTO’s TRIPS Agreement in 1994 (Dutfield, 2000; Andersen, 2008, 2016b).

In what has been described as the “Grand Bargain” (Gollin, 1993), the CBD laid down a new and unique approach to the exchange and use of genetic resources. In order to gain access to genetic resources, provider countries were required to consent to their use. In turn, users of genetic resources were required to provide fair and equitable benefits, including technology transfer, as agreed with providers. In order to receive such benefits, provider countries were required to facilitate access to genetic resources (hence “access and benefit sharing” or ABS) (Convention on Biological Diversity [CBD], 1992). In a similar vein, the use of traditional knowledge associated with these resources was to be recompensed through bilateral contracts and benefit-sharing agreements with holders of this knowledge. What the CBD and Nagoya Protocol meant in practice was that companies and signatory countries now had a legal obligation to get permission before collecting resources and knowledge (prior informed consent), mutually agree on the terms of exchange, and share benefits fairly with local providers and countries. User countries were also required to support compliance with the ABS regulations of provider countries. This highly transactional approach was largely reliant on contracts negotiated between so-called providers and users of genetic resources and traditional knowledge.

The CBD represented a fundamental change in the way in which genetic resources were exchanged and viewed: no longer were they seen as the common heritage of humankind, as countries increasingly asserted sovereign rights over their biological and genetic resources and control over their access. By establishing ABS as the main instrument for achieving the objective of “fair and equitable sharing of benefits” derived from the use of genetic resources, the CBD endorsed not only the possibility of IPRs on products, but also state ownership of genetic resources (Halewood et al., 2012; Bjørnstad and Westengen, 2019). As a result, both private companies and states were sanctioned through international law to enclose the genetic commons (Sievers-Glotzbach and Christinck, 2020).

This new norm, whereby farmers and traditional knowledge holders are to be rewarded on an individual or collective basis for genetic material obtained from their fields and used in commercial varieties and/or protected with IPRs, represents the “ownership” approach to genetic resource management (Andersen, 2017). The rationale is that such a reward system is necessary to enable the equitable sharing of benefits arising from the use of agrobiodiversity and to establish an incentive structure for the continued maintenance of this diversity. In this ownership approach the objective of fair and equitable benefit sharing is intrinsically linked to IPRs through ABS.

In 2001, following protracted negotiations, the ITPGRFA, commonly referred to as the Plant Treaty, was adopted (Food and Agriculture Organization (FAO), 2001), addressing issues relating to agricultural genetic resources that were not dealt with specifically within the CBD (Esquinas-Alcazar, 2005; Andersen, 2008; Lawson et al., 2020). Importantly, it recognizes the enormous contribution made by farmers in the conservation and development of PGRFA and states that this input constitutes the basis of food and agriculture production throughout the world^8^. Responsibility for the implementation of farmers’ rights, as they relate to the management of PGRFA, rests with national governments. The rights are not defined, but certain measures to protect and promote these rights are suggested: the protection of traditional knowledge, the right to participate in equitable benefit sharing and the right to participate in decision making at the national level. Also, any rights that farmers have to save, use, exchange and sell farm-saved seeds and propagating materials are addressed.

**FIGURE 1:**
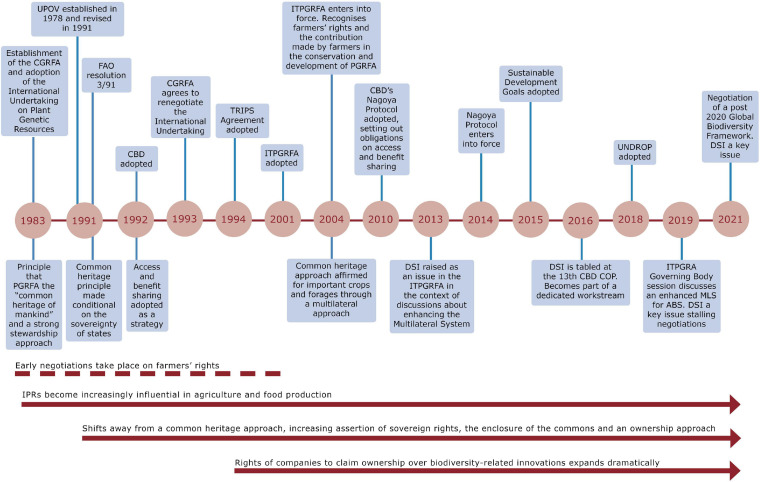
A timeline of key international agreements and trends shaping the ownership and use of PGRFA.

In contrast to the CBD, the Plant Treaty uses multilateralism to reaffirm a common heritage approach for 64 of some of the most important agricultural crops and forages (ITPGRFA, 2004; Andersen, 2008; Khoury et al., 2016). Implicit in this approach is the need to ensure uninterrupted germplasm flows for research and innovation, linked to the notion that the open accessibility of crop genetic resources has the potential to return benefits, such as improved crop varieties and scientific collaboration (Brush, 2007). To avoid some of the complicated contracting arrangements required by the CBD and Nagoya Protocol, the Plant Treaty has a Standard Material Transfer Agreement (SMTA) that has been used to distribute over 5.4 million samples (Food and Agriculture Organization (FAO), 2019b). However, while the FAO in a 2019 funding call for the Plant Treaty’s Benefit-Sharing Fund notes that voluntary payments have reportedly reached about one million farmers across 45 developing countries, with most being small-scale farmers, contributions made by users of PGRFA to the benefit-sharing fund established under the Treaty have been disappointing. Recently, there have been some positive developments, including the first user-based payments made to the fund by Nunhems Netherlands (a former subsidiary of Bayer and now of BASF), amounting to USD 119,083, and a decision by the French seed sector, Groupement National Interprofessionel des Semences et Plants (GNIS), to make regular annual voluntary contributions to the fund (Food and Agriculture Organization (FAO), 2019c). Nonetheless, a lack of progress on user contributions largely stimulated the commencement of negotiations to revise the MLS. Figure 1 provides a synthesis timeline of these key international agreements and trends that have shaped the ownership and use of PGRFA.

### The Emergence of DSI and Its Intersection With the Nagoya Protocol and the MLS of the ITPGRFA

Both the CBD and its Nagoya Protocol and the ITPGRFA and its MLS are based on the collection and exchange of physical material, although the ITPGRFA has been more forward-looking and also takes associated information into account in some of its provisions^9^. Indeed, in crop science there has been long-standing ambiguity as to whether the meaning of the term “genetic resource” includes both the physical material and/or the DSI (e.g., McLaren et al., 2005; Ishimaru et al., 2010; Holland, 2015). Negotiations for the Nagoya Protocol largely ignored the exponential growth in biotechnology (Wynberg and Laird, 2018), and its increasing reliance on the use of genetic sequence data and information found in databases (Laird and Wynberg, 2018). Whether or not the Nagoya Protocol includes DSI in its definition of the “utilization” of genetic resources, or merely as descriptive information out of its regulatory scope, is therefore now a matter of contention. In any case, DSI is accessed, valued, managed and used in very different ways to physical materials, suggesting that fundamental changes will be required to international legal frameworks to accommodate these new realities (Ruiz Muller, 2015; Laird and Wynberg, 2018; Rohden et al., 2019; Scholz et al., 2020; Aubry et al., 2021).

Table 1 summarizes some of the assumptions that underpin genetic resource use under the Nagoya Protocol and the MLS of the ITPGRFA, and compares them to the realities of DSI and the use of crop genetic diversity. For example, ABS under the Nagoya Protocol presumes that providers and users negotiate agreements and exchange physical material with clear provenance, ownership and value, but DSI turns most of this on its head, and changes the “rules of the game” entirely (Lawson et al., 2018; Laird et al., 2020). As noted by Aubry (2019), the modalities of DSI make traceability irrelevant, with the value lying in the amounts of data analyzed, rarely in a single accession. Moreover, DSI is typically valuable in the aggregate, with sequences shared across organisms and regions, and DSI changing throughout the research process. These and other factors make the bilateral ABS approach of the Nagoya Protocol a difficult if not impossible fit with DSI (Laird and Wynberg, 2018; Rohden et al., 2019; Scholz et al., 2020).

**TABLE 1:** Facts and fiction. Assumptions underpinning the Nagoya Protocol and the multilateral system of the ITPGRFA – and the reality of DSI and the use of crop genetic diversity.

Assumptions underpinning the Nagoya Protocol	Assumptions underpinning the multilateral system of the ITPGRFA	The reality of DSI And The Use Of Crop Genetic Diversity
**Access to material**		
Genetic resources are primarily physical material accessed *in situ* and, to a lesser extent, through *ex situ* collections.	Genetic resources are accessed mostly through public and private *ex situ* collections.	Most DSI is accessed through databases, although some might be sourced through *in situ* or *ex situ* collections.^i^
**Determining value**		
Discrete products and processes can be tracked and valued based on the genetic resources used.	Valuation is challenging due to the fact that the innovation process in breeding is typically of an incremental nature.^ii^	The value lies in the amounts of data analyzed, in aggregate, rarely in a single accession. However, the multiple sources and organisms that comprise DSI make valuation near impossible.^iii^
**Identifying providers and users**		
It is possible to identify providers and users of genetic resources, and thus require and negotiate ABS agreements.	All countries are interdependent on PGRFA. Parties to the ITPGFRA provide crop resources to the MLS which is in the public domain and under their control. Users can access the material by signing a SMTA, which establishes the terms and conditions for use and benefit sharing.	Most DSI is accessed through public databases which for the most part do not require providers and users to provide metadata about provenance and use.^iv^
**Identifying the provenance of DSI**		
Genetic sequence data can be linked back to the original physical material.	The origin of genetic resources is highly convoluted due to millennia of cross-border transfers, multiple parental sources, and the variety of location-specific traits that are acquired.	Although there are increased efforts to link the original physical material with DSI, this may not always be possible.^v^
**Monitoring use**		
By tracking use, benefits can be determined and fairly shared.	The incremental R&D process makes it challenging to track the movement of genetic resources through different value chains and geographical locations. A complicated system to track transfers would hamper expeditious access.^vi^	Sequences are notoriously difficult to monitor as they pass through multiple hands, are modified through the research process, and also change identity over time.^vii^
**Benefit sharing**		
Benefit sharing is primarily bilateral although Article 10 of the Nagoya Protocol proposes a multilateral benefit-sharing mechanism. Bilateral benefit-sharing arrangements have yielded valuable non-monetary benefits over time, but few financial benefits. Negotiation of these agreements has proven lengthy, complex, and an obstacle to both academic and commercial research.	The MLS facilitates benefit sharing through the SMTA and is regarded as a benefit in itself. Those who access and develop genetic materials through the MLS agree that they will either freely share any new developments for further research or pay a percentage of any commercial benefits into a common fund to support farmers in developing countries. Benefit sharing is often complex due to the cumulative nature of plant breeding; because R&D leading to the final product may require exchanges that do not take place within one company; and because intermediate products themselves are sometimes marketed.^viii^	Possibilities exist to delink access to specified sequences from benefit sharing and to use a subscription or differentiated fee-based system for DSI.^ix^ Implementing a system for sharing monetary benefits based on specified sequences is highly complex due to the contribution of sequences from multiple species, sources, pathways and producers. Non-monetary benefit-sharing opportunities also exist such as capacity development and research collaborations.
**Commercial and non-commercial research**		
Although the system allows for a distinction to be made between commercial and non-commercial research, such differences are often difficult to establish.	Material in the MLS is available for research and breeding under the SMTA but if commercialized benefit sharing may be required.	Lines between research activities are increasingly indistinct as DSI moves fluidly between commercial and non-commercial institutions.^x^

*^*i*^Laird and Wynberg, 2018; Rohden et al., 2019.*

*^*ii*^Fowler and Hodgkin, 2004; Smolders, 2005.*

*^*iii*^Houssen et al., 2020; Laird et al., 2020; Aubry et al., 2021.*

*^*iv*^Bagley, 2017; Rohden et al., 2019.*

*^*v*^Laird and Wynberg, 2018; Houssen et al., 2020.*

*^*vi*^Fowler and Hodgkin, 2004; Esquinas-Alcazar, 2005.*

*^*vii*^Garrity et al., 2009; Scott and Berry, 2017; Laird and Wynberg, 2018; Rohden et al., 2019; Scholz et al., 2020.*

*^*viii*^Smolders, 2005.*

*^*ix*^Welch et al., 2017; Lawson et al., 2020; Scholz et al., 2020.*

*^*x*^Laird and Wynberg, 2018.*

However, many of the features of DSI align well with the MLS, which recognizes the challenges of valuing incremental innovation, of identifying the provenance of PGRFA, and of tracking the movement of genetic resources through different value chains. Recognition by the MLS of the interdependence of countries on PGRFA also reflects the manner in which DSI is accessed globally through public open access or open source databases (Khoury et al., 2016). Finally, the subscription model that was under deliberation by the Governing Body of the ITPGRFA when negotiating revisions to the SMTA, envisages user-based payments to the MLS, and could tally well with proposals to charge levies or membership fees for the use of DSI as an approach to monetary benefit sharing more in sync with the realities of user patterns for DSI (Welch et al., 2017; Lawson et al., 2020; Scholz et al., 2020). The next section uses the stewardship/ownership lens to compare and contrast what these different aspects mean for the relationship between farmers’ rights and DSI.

## Farmers’ Rights and DSI

### DSI and the Protection of Traditional Knowledge as a Measure to Promote Farmers’ Rights

Over the last ten millennia, farmers from across the cultivated regions of the world have, through collective action, contributed to developing the enormous diversity of crops that is available today and which provides the foundation of food and agriculture (Brush, 2007; Desmarais, 2007; Andersen, 2016b). By utilizing local knowledge passed on for generations, farmers have selectively bred plants and animals that not only meet their needs and preferences, but are also adapted and adaptable to changing ecological conditions and local climates. Through age-old customs such as saving and exchanging seed, farmers have contributed, and continue to do so in many parts of the world, to the spread and diversification of germplasm (van Dooren, 2008).

Article 9.2a of the ITPGRFA affirms the importance of this traditional agricultural knowledge and states that parties shall take measures to protect traditional knowledge relevant to PGRFA. While about 90–95% of all genetic resources used today in plant breeding are elite, modern varieties, derived from private genebanks, with only the remaining 5–10% representing landraces or wild relatives, there is growing interest and investment in utilizing crop wild relatives and farmer varieties (Smolders, 2005; Baldermann et al., 2016; Dempewolf et al., 2017; Aberkane et al., 2019; Kilian et al., 2020; Singh et al., 2020). This attention is due in part to the fact that they contain important genes for stress resistance, adaptability, and improved productivity, and are therefore of interest in the context of climate change, population growth, shrinking areas of arable land and the rapid erosion of agrobiodiversity (Dempewolf et al., 2017; Aberkane et al., 2019; Kilian et al., 2020). Changes in consumer demand are also transforming the interest in crop wild relatives and underutilized species, with consumer interest in novel and ‘super’, or highly nutritious foods growing in recent decades (Wynberg, 2013). In addition, there is increasing awareness of the potential of improving farmers’ varieties, local land races and crop wild relatives for local use through participatory plant breeding schemes, due to their adaptability to local environmental conditions, nutritional values and consumer preferences (e.g., Andersen, 2019; Westengen and Winge, 2020).

Developments in genomics and molecular biology are likely to enhance the characterization and evaluation of wild genetic resources and landraces, and hence the DSI publicly available. If farmers have reared a particular plant to express desired traits over generations and this plant’s genome is sequenced, then the traditional knowledge of those who bred this plant is embedded within this DSI – although some species will clearly be more actively bred and managed than others (Smyth et al., 2020).

A central question, therefore, is whether traditional knowledge can be decoupled from a plant’s underlying genomic information, should it be transcribed into DSI. Identifying links to traditional knowledge within sequences is challenging given that genetic resources are drawn from multiple sources and organisms, may include repetitive stretches of DSI, typically do not include provenance data, and may change during the research process (Laird and Wynberg, 2018; Houssen et al., 2020; Scholz et al., 2020). It is therefore unlikely that traditional knowledge from the DSI of a sequenced plant can be legally, politically or technically dissociated (Smyth et al., 2020). Thus there is a concern that “companies and others can obtain traditional knowledge through publications, interviews, or other means and then undermine indigenous peoples’ control over the physical genetic resource by deriving genetic information and recreating key genes from DSI instead of signing an access agreement” (African Center for Biodiversity [ACB], 2020).

An ownership approach to this concern would emphasize the risk of misappropriation of genetic resources and associated traditional knowledge and hence the need to regulate DSI accordingly. From this perspective, avoiding “digital biopiracy” would mean that farmers have the right to act against such misappropriation and to decide how their knowledge and related plant genetic resources could be used. Traditional knowledge and local customary rights over genetic resources would, according to this approach, be “regularized” using the dominant, rights-based intellectual property regime (West, 2012).

A stewardship approach, in contrast, would protect farmers’ traditional knowledge against its ongoing and substantial decline, seeking to keep it alive and develop it further (Andersen, 2016a). This might be by promoting the sharing of farmers’ varieties, promoting participatory plant breeding, reviving traditional methods of storage, strengthening cultural ties to land and/or support for traditional practices in younger members of communities, and supporting land and resource rights and respect for customary law. In this “protection by sharing” mode, the unrestricted sharing of DSI would be unproblematic, as long as IPRs did not negatively impede the ongoing use and exchange of traditional varieties.

Both approaches share concerns about the potential negative effects on farmers’ rights of IPRs acquired by external actors, but differ as to how significant this potential threat might be. A potential compromise might lie in establishing *prior art*, i.e., by documenting relevant PGRFA and associated traditional knowledge in such a way that it cannot be made subject to IPRs in its existing form.

### DSI and Farmers’ Rights to Participate in Benefit Sharing

The rights of farmers to participate equitably in the sharing of benefits arising from the use of PGRFA is a central measure in the ITPGRFA^10^. Article 13, referring to the MLS, includes such benefits as (1) facilitated access to plant genetic resources for food and agriculture; (2) the exchange of information; (3) access to, and transfer of, technology; (4) capacity building, and (5) the sharing of monetary and other benefits arising from commercialization. Benefits shared under the MLS are intended to flow to all farmers, but especially to those in “developing” countries and economies in transition, who conserve and sustainably utilize PGRFA. Benefit-sharing measures can be designed in many ways, with the ownership and stewardship approaches both providing insights as to how DSI might be managed to realize this aspect of farmers’ rights.

Under an ownership approach, the focus is typically on monetary benefits arising from commercialization. Such benefit-sharing agreements are negotiated on a bilateral basis between the purported “providers”/ “owners,” and “users” of genetic resources – based on prior informed consent and mutually agreed terms, as stipulated by the CBD and its Nagoya Protocol. However, despite comprehensive efforts over the past three decades, there are few examples of monetary benefit sharing between providers and recipients of PGRFA through this approach (West, 2012; Andersen, 2016a, 2017; Peschard and Randeria, 2020). Where they do exist, evidence suggests that these “new property-based schemes for farmers and communities are unworkable and likely to forestall more viable approaches to address the needs of conserving genetic resources and improving rural livelihoods” (Brush, 2007; West, 2012). Including DSI in the CBD architecture is unlikely to improve the effectiveness of benefit sharing and may well restrict access and use of DSI and impede innovation, while at times working against the interests of farmers and Indigenous and local communities (Gaffney et al., 2020; Laird et al., 2020; Scholz et al., 2020). Remarks Kloppenburg (2014, 1237), “compensationist approaches to ‘access and benefit sharing’ have neither protected farmers and Indigenous peoples from biopiracy nor brought them any benefit, but have functioned mostly to legitimate and institutionalize their continued expropriation.”

In contrast, the stewardship approach inspires more effective benefit sharing (Andersen and Winge, 2013), growing from the early negotiations on farmers’ rights in the 1980s (Andersen, 2005, 2016b). A basic principle established at that time was that benefits should be shared among “entire peoples,” the stewards of plant genetic resources in agriculture and society at large (Food and Agriculture Organization (FAO), 1987, Appendix F, section 8). This principle is based on the idea that farmers have a right to be rewarded for their contributions to the global genetic pool from which we all benefit, and that the international community is obliged to ensure that such rewards are provided. It recognizes that knowledge and use of agricultural genetic resources is often shared widely across communities and groups. This is also the approach upon which the MLS and its benefit-sharing fund is founded. Benefit sharing may also take other pathways, such as through development cooperation (Brush, 2007; Andersen, 2008).

Embracing a stewardship approach would enable the use of DSI to contribute to benefit sharing in multiple ways. In addition to monetary benefits through a subscription system or through other means, it may spur innovation in plant breeding and thus provide farmers with better and more adapted crop varieties. The advantages associated with DSI through bioinformatics may also support participatory plant breeding and help support farmers’ needs more effectively. Wider access to databases, knowledge and technology, as well as research directed toward the much-neglected needs of small-scale farmers (Wynberg et al., 2018) are additional forms of benefit that could arise from DSI (Laird and Wynberg, 2018). The wider availability of DSI through open access to and exchange of DSI is also regarded by many as a significant benefit (e.g., Gaffney et al., 2020), although the differential capacities and resources of researchers in the global North and South to “access, analyze and finally publish” DSI raise important questions of equity and fairness (Aubry et al., 2021). Capacity building, technology transfer and infrastructure support are clearly critical to address these uneven scientific capacities.

Both the stewardship and ownership approaches, however, bring challenges for sharing monetary benefits from the use of DSI, due to the difficulties of identifying provenance^11^ and the value of any given sequence. Moreover, benefits arising from the use of DSI are typically deferred to a point in the future when a commercial product has been developed, involving the negotiation of monetary benefits through database and registry conditions of use notices, MTAs, licenses and user agreements (Laird and Wynberg, 2018). Resolving this conundrum requires a delicate balance between supporting access to DSI while ensuring fair and equitable benefit sharing for farmers in the global South (Laird and Wynberg, 2018).

Proposed DSI access fees or annual subscriptions (Welch et al., 2017; Scholz et al., 2020; Lawson et al., 2020), could be linked to the creation of a new fund, or to an existing fund, such as the benefit-sharing fund under the MLS. It is noteworthy that the subscription system that has been under consideration by the Governing Body of the ITPGRFA, has the potential to enable such an approach, possibly replacing the SMTA as the primary means of accessing the MLS (Rabitz, 2017). Also important to note are parallel discussions within the Governing Body about the timing of payments and the nature of voluntary obligations, both of which have implications for the way in which the fund could operate to ensure that benefits accrue to support farmers’ needs in the global South.

### DSI and Farmers’ Rights to Participate in Decision-Making

The rights of farmers to participate in national decision making on matters related to the conservation and sustainable use of PGRFA is a third suggested measure for the realization of farmers’ rights^12^. There are several preconditions that would enable farmers to be more actively engaged in decision making. First, the role played by farmers in conserving and developing PGRFA would need to be politically recognized, enabling a democratic, inclusive and accessible space for farmer voices to be heard and their engagement in policy formulation to hold weight. Small-scale farmers in particular remain marginalized in many policy processes, and their lack of economic power often undermines the positions they may hold. Asymmetries in power due to the dominance of the private sector in agricultural policy spaces are relevant in this regard (Desmarais, 2007; Clapp, 2018). Second, decision makers would need to be aware of the role of farmers in contributing to national food security, in order to understand why their participation is important. Third, capacity building is a precondition to enable farmers to participate effectively in decision-making processes that are often convoluted, bureaucratic and inaccessible. And fourth, the value of farmer-led innovations and knowledge would need to be emphasized and recognized in decision-making about agricultural research and development. This would entail the inclusion of farmer-based knowledge systems in agricultural research and development and the active participation of farmers from the global South in defining priorities and undertaking research and development that responds to local needs (United Nations [UN], 2018).

Digital sequence information (DSI) makes these decision-making processes even more challenging. With resources and information dislocated, and farmers’ knowledge submerged in nucleotide databases at the other end of the world, farmers’ rights, knowledge and their participation in decision-making may well seem like a parallel universe. While neither the ownership nor stewardship approach offer clear solutions in this regard, the stewardship approach would provide the legal space for farmers to continue their practices as custodians and innovators of PGRFA and would enable a more inclusive, participatory and deliberative space for farmer engagement.

### DSI and Farmers’ Rights to Save, Use, Exchange, and Sell Farm-Saved Seed

The rights that farmers have to save, use, exchange and sell farm-saved seed,^13^ remain one of the most contentious issues in international and national agricultural policy and law. Proponents argue that these rights are crucial to the continued contribution of farmers to the conservation and sustainable use of crop genetic resources, whereas critics claim that such rights need to be restricted to safeguard innovation in commercial plant breeding and to ensure that plant health and seed quality are guaranteed in seed distribution (e.g., Borowiak, 2004; De Jonge et al., 2015; Westengen, 2017). Despite recognition that both commercial and farmer-led seed systems are required to contribute to the conservation and sustainable use of crop genetic diversity, the policy space that they occupy remains contested. Evidence suggests that IPR laws which promote plant breeder’s rights and seed laws that regulate variety release and seed distribution in many countries may well be prejudicial to the interests of small-scale farmers, restricting the legal space they have to continue customary practices (Andersen, 2008; West, 2012; Andersen, 2017).

Increased use of DSI in plant breeding could escalate IPRs over PGRFA, in terms of plant variety protection as well as patents. This could further restrict the legal space for farmers to save, use, exchange and sell farm-saved seed of protected varieties. The greatest danger in this regard is patents which cover the properties of plants and which may extend to local and traditional varieties.

An ownership approach would not solve these challenges but could provide farmers with the possibilities to formally register their plant varieties and to obtain IPRs on varieties they develop on an equal footing with breeders. The use of DSI would have no implications in this regard but, rather than leading to a realization of farmers’ rights by maintaining systems of free exchange, would incorporate traditional farmers into the IPR system (West, 2012).

In contrast, the goal under a stewardship approach would be to grant rights to save, use, exchange and sell farm-saved seed by securing the legal space for such customary practices. The implementation of ‘biological open-source’ arrangements could undergird such a stewardship approach and link to the creation of a protected commons populated by farmers and plant breeders. This could see materials freely available and widely exchanged, but “protected from appropriation by those who would monopolize them” (Kloppenburg, 2010, 367).

Of interest, is the parallel development of open source approaches in DSI databases, which create a “contractually constructed research commons” that allows DSI research, which is dependent upon exchange, collaboration, and the free flow of information, to flourish in highly protectionist intellectual property environments (Reichman and Okedji, 2012; Reichman et al., 2016). As Laird and Wynberg (2018) describe, these approaches are intended to facilitate the free exchange of information, technology and materials, and support increasingly networked and collaborative research. They also allow for greater transparency and visibility. Users are required to join a community through an agreement that attaches some conditions, in exchange for rapid and easy exchange of materials and sequences, allowing for a form of technology-transfer within the research community (Lawson and Rourke, 2016; Elbe and Buckland-Merrett, 2017). Contributors may request attribution and reporting for materials, but IPRs are typically not asserted against materials if the conditions of the open source license are met, and may also be transferred between researchers within the open source community, whether academic or commercial. Some agreements require that anything developed from materials be shared with the community of contributors and users, but others do not, and none include royalties for the use of materials or methods.

## Discussion. A Stewardship Approach for Farmers’ Rights and DSI

The digitization of genetic resources has brought the management of crop diversity into sharp focus and, arguably, places the realization of farmers’ rights at a crossroads. It also comes at a time of great transition and retrospection in the history of agriculture and food production, as awareness grows of the negative environmental impacts of industrial agriculture, including on biodiversity and forests, its large climate footprint and the related crises of food and nutritional security (Campbell et al., 2017; Pereira et al., 2018; Altieri and Nicholls, 2020). The COVID-19 pandemic has intensified these issues, underpinning the need for fundamental changes to our food and production systems, at the same time emphasizing the critical role played by the world’s 1.5 billion smallholders, family farmers and their knowledge systems (Altieri and Nicholls, 2020; Clapp and Moseley, 2020). In parallel, contestations over DSI in multilateral agreements dealing with ABS, remain unresolved and a significant stumbling block toward finding resolutions for biodiversity, health and food security goals.

The interrelatedness of these issues is undeniable. We suggest that despite implementation challenges, the time is ripe for opening doors to finding fair and equitable solutions beyond existing ABS approaches – not only for DSI governance but also for the management and use of PGRFA and the recognition of farmers’ rights.

This paper set out to open questions about the implications for farmers’ rights, as recognized in the ITPGRFA, of the dematerialization of genetic resources. We showed how the intensifying use of DSI for crop genetic resources poses new possibilities for the realization of farmers’ rights, as well as increased risks and threats. In this regard, we described two points of departure: (a) an ownership approach, that aligns with the ABS framing of the CBD, and which is characterized *inter alia* by the enclosure of rights through the use of IPRs; and (b) a stewardship approach, based on common heritage principles (see Table 2 for a summary of these approaches).

**TABLE 2:** Comparing Stewardship and Ownership Approaches for Farmers’ Rights and DSI.

	Ownership approach	DSI implications	Stewardship approach	DSI implications
**Protection of farmers’ traditional knowledge (Art. 9.2b)**	Aims to protect farmers’ knowledge from misappropriation and to enable knowledge holders to make decisions over its use.	DSI related to traditional knowledge would only be shared with prior informed consent on mutually agreed terms.	Aims to protect farmers’ knowledge from further erosion and thus to encourage its further use.	Unrestricted sharing of DSI may increase the use of traditional varieties, provided that IPRs can be avoided.
**The right to participate in equitable benefit sharing (Art. 9.2b)**	Benefits shared between purported ‘owners’/providers and ‘buyers’/users of genetic resources upon prior informed consent on mutually agreed terms.	Access to DSI only provided if equitable participation in benefit sharing can be guaranteed on mutually agreed terms.	Benefits shared between stewards of crop genetic resources and wider society through the MLS, national support schemes, and development cooperation.	The use of DSI may contribute to benefit sharing by innovation in plant breeding and participatory plant breeding, capacity building, technology transfer, and infrastructure support.
**The rights to participate in decision-making at the national level(Art. 9.2c)**	Participation is important to ensure adequate legislation on ABS and intellectual property rights.	Participation is important to ensure that DSI is covered effectively in ABS legislation.	Participation is important to facilitate a legal space and to enable rewards for farmers’ contributions to the genetic pool.	Participation is important to ensure regulation on DSI that safeguards a legal space for farmers to maintain their genetic resources.
**Any rights that farmers have to save, use, exchange, and sell farm-saved seed(Art. 9.3)**	Aims to balance the rights of farmers with plant breeders’ rights and other forms of IPR.	Aims to enable farmers to utilize DSI on an equal footing with breeders, with equal access to IPR.	Aims to uphold or enhance a legal space to ensure that farmers continue to maintain plant genetic resources as a basis for food security.	Aims to ensure that the use of DSI does not impede the legal space for farmers.

*Adapted from Andersen (2016a).*

We illustrated how an ownership approach focused on regularizing the use of traditional knowledge through ABS approaches and IPRs, could introduce restrictions on the sharing of DSI. We also revealed that an ownership approach to the management of crop genetic resources enables different actors to exclude each other from access to, and the use of, these vital resources, thereby also reducing the legal space for all to contribute to the conservation and sustainable use of crop genetic diversity (Andersen, 2008). The ownership approach also poorly accommodates the fact that the exchange of PGRFA is most often part of longer term and broader collaborations (Louafi and Welch, 2021) (Table 2).

Efforts to fold DSI into ABS provisions stem from concerns that the entire ABS framework will not function if a significant portion of material falls outside its scope. For example, new molecular tools and approaches are increasingly leading to better understanding of molecular processes, allowing for greater precision in the identification of genes for crop improvement (Tanksley and McCouch, 1997; Spindel and McCouch, 2016; Dempewolf et al., 2017; Halewood et al., 2018). Whole genome sequencing is revolutionizing analysis of crop germplasm, and is increasingly used to identify traits in breeding programs (Manzella, 2016; Yu et al., 2016; Milner et al., 2019; Sansaloni et al., 2020). Plant genomic information is also being mined to identify genes of interest, which may be used to edit agricultural crop genomes as well as applications outside of agriculture (Welch et al., 2017).

An ownership approach to governing DSI runs counter to the practice and ideals of this burgeoning publicly oriented science (e.g., Wilkinson et al., 2016)^14^ and the way in which DSI is used and managed, and science practiced today. For example, in order to publish molecular scientific results in reputable academic journals, authors must make the data available for others to scrutinize and build on in further research^15^. Adopting DSI governance regimes in international biodiversity agreements that are at odds with these principles is likely to render the agreements less relevant and create further animosity in a scientific community already hostile to the bureaucracy and confusion created by the Nagoya Protocol and its continued use of expensive checkpoints and oversight (e.g., Neumann et al., 2018; Prathapan et al., 2018; Laird et al., 2020). As Lawson et al. (2020, 32) describe: “put simply, ABS under the CBD, Nagoya Protocol, Plant Treaty and the PIP Framework is an enclosure of what previously has been available with few to no restrictions. Enhancing the existing contract context to include information about genetic resources as a resource derivative within the ABS transaction itself is expanding this enclosure further, and beyond the original ideal of open access and information sharing.”

In contrast to the ownership approach, our analysis reveals that a stewardship approach could protect farmers’ traditional knowledge against further decline by increasing the use of traditional varieties in applications tailored toward farmers’ priorities. A stewardship approach would also maintain and enhance the legal space and possibilities to contribute to the conservation and sustainable use of crop genetic resources by supporting traditional agriculture, including the use and free exchange of farmers’ seed. The paradox, however, is that without sufficient measures, this approach could result in genetic resources and associated information being privatized, with ownership captured by those other than farmers and knowledge holders. In this regard, we suggest that open-source arrangements might hold potential to create a protected commons populated by farmers and plant breeders whose materials would be freely available and widely exchanged but protected from appropriation.

The question arises as to how stewardship can be secured within a framework that achieves conservation and sustainable use of genetic resources, the sharing of benefits arising from the use of these resources as well as associated information, and the realization of farmers’ rights, while integrating the rapid advances in science and technology that are changing the ways genetic resources are used. For example, today many new crops are developed using DSI that is not from plant genetic resources, and synthetic biology is an increasing area of research focus. As Beumer et al. (2021) describe, institutions impact innovations, but scientific and technological innovations also impact institutions. They propose “the perspective of *co-production* as a fruitful way to understand the interaction between technological innovation and the commons” (Beumer et al., 2021). If we adopt this framing, it may be that we find ourselves in a time of rapid co-production of policy formulation as ABS is reconsidered and reconceived in light of DSI (Laird et al., 2020).

## Actionable Recommendations: Pathways to the Governance of DSI

As part of this co-production process, we suggest that a stewardship approach to understanding farmers’ rights provides a platform for framing the DSI discourse, and as such may help in finding solutions for addressing DSI within policy frameworks intended to promote equity and conservation. Based on the stewardship approach, we thus propose two possible complementary pathways that could be followed in opening up new ways to understand and govern DSI. The first pathway envisages an enhanced MLS system based on a subscription approach that includes DSI and which promotes and enhances the realization of farmers’ rights. The second, more far-reaching proposal is what we call an “expansive” stewardship approach, folding together concepts of stewardship, farmers’ rights, and open source science.

### Pathway 1: Stewardship Within an Enhanced MLS

The first pathway we describe proposes enhancing the functioning of the benefit-sharing mechanism under the MLS, linked to previous proposals tabled in the Governing Body of the ITPGRFA. This would require developing a fair, equitable and efficient system for monetary benefit sharing based on a subscription approach that includes DSI and which promotes and enhances the realization of farmers’ rights. This option would entail a multilateral solution to the governance of DSI and would include measures to ensure that IPRs on new crops developed with the use of DSI do not pose barriers to the further sharing and use of the DSI and related physical crop genetic resources and traditional knowledge used for the innovation. It would also require capacity building and technology transfer to enable public plant breeders and those engaged in participatory plant breeding to effectively utilize DSI.

While this would be a major step forward, it may not go far enough. As Clapp (2018, 28) points out, “existing regulatory and institutional frameworks are weak and disjointed,” while Mytelka (2000) reminds us that corporate agribusiness actors have considerable power to shape political structures and thus hinder any obstructions to their business. Moreover, although the MLS conforms in many respects to what is referred to as a “new commons” (Halewood, 2013), the system has been met with critique and skepticism both from civil society organizations and the private sector (Halewood et al., 2012; Halewood, 2013; African Center for Biodiversity [ACB], 2019; Peschard and Randeria, 2020)^16^. Seemingly agreeing with critical civil society perspectives, Kloppenburg (2014, 1236) retorts, “20 years of struggle over the form of the treaty produced little more than an affirmation of the primacy of intellectual property rights.”

### Pathway 2: Undergirding Stewardship Through Open Source Arrangements

A parallel and complementary but more ambitious pathway could explore an overarching “expansive stewardship” approach, folding together concepts of stewardship, farmers’ rights, and open source science and breeding. Such approaches are already beginning to take shape and could develop into a sizeable alternative. Multiple initiatives are emerging across the world to introduce these more innovative and democratic ways of working, based on collaborations to share knowledge and seed that are unencumbered by property rights and other restrictions. In Argentina, for example, Bioleft^17^ aims to “develop and redistribute collective agency over seed breeding, as a response to the emergence of an oligopolistic seed industry” by creating an open-source, networked approach to breeding that supports the production needs of small farmers, and those working within other low-input agricultural systems (Cremaschi and van Zwanenberg, 2020). Similarly, in the United States and other countries (Luby et al., 2015), an Open Source Seed Initiative has been launched to apply legal mechanisms from the open source software movement to plant breeding (Kloppenburg, 2014). The intention, as articulated by Kloppenburg (2014, 1243) is to reconstitute the commons by creating a “positive, relatively autonomous space in which capital might be effectively prohibited – by its own rules – from trespassing.” In this way, processes of dispossession will not only be impeded, but might actually facilitate the repossession of ‘seed sovereignty’. Open source licenses preserve the right to use material for breeding and the rights of farmers to save and replant seed, and there is an explicit rejection of payment for access to genetic resources and benefit sharing based on the dependency of this model on enclosure, profit and commodification.

Such innovations, which reflect new forms of social and productive organization based on norms of sharing and solidarity (Cremaschi and van Zwanenberg, 2020), also align well with the United Nations Declaration on the Rights of Peasants and Other People Working in Rural Areas (UNDROP), adopted by the UN General Assembly in 2018 (United Nations [UN], 2018). Although the Declaration is not legally binding, it aspires for individual and collective rights to be granted to local communities for land, seed and natural resources and for research priorities both to be defined and implemented by farmers^18^ (United Nations [UN], 2018).

Despite these opportunities, much work remains in finding approaches that forge the gap between the material realities of seed, land and capital and the digital realities of the scientific and breeding community who access and use DSI. As Cremaschi and van Zwanenberg (2020) remark, the social and cultural contexts of farmers are often far removed from digital infrastructure and the success of open-source software is unlikely to be straightforward to replicate. Moreover, farmers and scientists have very different worldviews and knowledge systems (De Wit, 2016), and the dominance of modernist science would need to be recognized and addressed in the search for common solutions, creating space for other ways of knowing about agrobiodiversity (van Dooren, 2008).

## Conclusion

Contestations about the way in which DSI is used and regulated have created stumbling blocks across multiple international policy processes and have profound implications for the way in which we manage and conceptualize agrobiodiversity and its benefits. At the same time, there is a clear need to move away from viewing PGRFA as a commodity that can be owned, toward a strengthened, proactive and expansive stewardship approach that recognizes PGRFA as a public good which should be governed as such. These imperatives present synergistic opportunities to find solutions for addressing DSI within policy frameworks intended to promote equity, conservation and sustainable use through farmers’ rights. Two possible and parallel pathways are presented, the first envisaging an enhanced MLS system that includes DSI and which promotes and enhances the realization of farmers’ rights; and the second a more radical approach that folds together concepts of stewardship, farmers’ rights, and open source science. Both imagine a “reconstituted commons” (Kloppenburg, 2014), both firmly embed farmers from the global South in an access and benefit-sharing solution that is developed for DSI, and both require Indigenous communities and farmer worldviews to be brought into sharp focus and prominence. Whereas the first pathway of an enhanced MLS would need to be negotiated and agreed at the international level, the second pathway toward open source arrangements is already evolving and may continue to do so as an emerging alternative to international regimes relating to genetic resources and DSI. DSI as a crisis could well be seized at this critical policy juncture as an unexpected but serendipitous opportunity to chart an alternative and visionary pathway for the rights of farmers and other custodians of plant genetic resources.

## Author Contributions

RW wrote the first draft of the manuscript and finalized all revisions. RA, SL, and OW wrote sections of the manuscript. All authors contributed to the conceptualization and design of the study, revised drafts, read, and approved the submitted version.

## Conflict of Interest

The authors declare that the research was conducted in the absence of any commercial or financial relationships that could be construed as a potential conflict of interest.

## Publisher’s Note

All claims expressed in this article are solely those of the authors and do not necessarily represent those of their affiliated organizations, or those of the publisher, the editors and the reviewers. Any product that may be evaluated in this article, or claim that may be made by its manufacturer, is not guaranteed or endorsed by the publisher.

## Footnotes

1 Digital Sequence Information (DSI) is not a term typically used by the scientific community but has become adopted as a placeholder for negotiations. Terms more commonly employed include *genetic sequence data, nucleotide sequence data, nucleotide sequence information*, and *genetic sequences*. Differences in terminology in scientific circles reflect differences in the material referred to, as well as the speed and transformative nature of technological change today, which make it difficult to harmonize terminology (Laird and Wynberg, 2018). In ABS policy discussions, differences in terminology often reflect divergent views about what falls within the scope of the Nagoya Protocol and national laws, ranging from the inclusion of DNA and RNA sequences, through to protein sequences and their resulting metabolites (AHTEG, 2020).

2
https://www.foodsovereignty.org/the-ipc/

3 See, for example the submissions from Canada and the United States available at http://www.fao.org/plant-treaty/overview/mypow/dsi/en/).

4 There are more than 1,500 publicly accessible biological databases, organized based on heterogeneity, data type, scope and curation. The largest databases are part of the International Nucleotide Sequence Database Collaboration (INSDC). This is comprised of three global partners: The European Nucleotide Archive, based at the EMBL European Bioinformatics Institute (EMBL-EBI) in Cambridge, United Kingdom; GenBank, based at the National Center for Biotechnology Information (NCBI) in Bethesda, Maryland, and the DNA Data Bank of Japan (DDBJ), based at the National Institute for Genetics in Mishima, Japan.

5 WTO TRIPS https://www.wto.org/english/tratop_e/trips_e/intel2_e.htm.

6 UPOV https://www.upov.int/portal/index.html.en.

7 The total value of purchased seeds in 2014, excluding farm-saved seed but including public commercial varieties.

8 Article 9.

9 See, for example, Articles 5, 12, 13, 14, and 17.

10 Article 9.2b.

11 Although researchers and databases increasingly embrace the inclusion of provenance and meta-data, over half of DSI that does identify the country of origin comes from the United States, China, Canada and Japan rather than from countries of the global South (Rohden et al., 2019). A benefit-sharing scheme linked to provenance would, ironically, thus provide substantial benefits to the global North at present.

12 Article 9.2c.

13 Article 9.3.

14 The FAIR principles, for example – Findability, Accessibility, Interoperability, and Reusability – put specific emphasis on enhancing the ability of machines to automatically find and use data, in addition to supporting its reuse by individuals. See also https://www.coalition-s.org/describing Plan S, an initiative for Open Access publishing.

15 https://www.nature.com/nature-research/editorial-policies/reporting-standards; https://www.sciencemag.org/authors/science-journals-editorial-policies

16 See also various statements by La Via Campesina https://viacampesina.org/en/viith-session-international-seed-treaty-lets-not-sweep-peasants-rights-seeds-carpet/ and the International Planning Committee for Food Sovereignty https://www.foodsovereignty.org/.

17
https://www.bioleft.org/

18 Article 19.

## Abbreviations

ABS: Access and benefit sharing
CBD: Convention on Biological Diversity
CGRFA: Commission on Genetic Resources for Food and Agriculture
COP: Conference of the Parties
DSI: Digital Sequence Information
GATT: General Agreement on Tariffs and Trade
INSDC: International Nucleotide Sequence Data Collaboration
IPC: International Planning Committee for Food Sovereignty
IPRs: Intellectual Property Rights
ITPGRFA: International Treaty on Plant Genetic Resources for Food and Agriculture
MLS: Multilateral System of the International Treaty on Plant Genetic Resources for Food and Agriculture
PGRFA: Plant Genetic Resources for Food and Agriculture
PVP: Plant Variety Protection
SMTA: Standard Material Transfer Agreement
TRIPS: Trade Related Aspects of Intellectual Property Rights Agreement
UNDROP: United Nations Declaration on the Rights of Peasants and Other People Working in Rural Areas
UPOV: International Union for the Protection of New Varieties of Plants convention
WTO: World Trade Organization.
